# Hantavirus Pulmonary Syndrome in Argentina, 1995–2008

**DOI:** 10.3201/eid1612.091170

**Published:** 2010-12

**Authors:** Valeria P. Martinez, Carla M. Bellomo, María Luisa Cacace, Paola Suárez, Liliana Bogni, Paula J. Padula

**Affiliations:** Author affiliations**:** Administración Nacional de Laboratorios e Institutos de Salud “Dr. C. G. Malbrán,” Buenos Aires, Argentina (V.P. Martinez, C.M. Bellomo, P.J. Padula);; Hospital San Vicente de Paul de la Nueva Orán, Salta, Argentina (M.L. Cacace);; Hospital Descentralizado–Región Sanitaria XI–Ministerio de Salud de la Provincia de Buenos Aires, Buenos Aires (P. Suarez);; Área Programática Esquel–Secretaría de Salud de la Provincia de Chubut, Esquel, Argentina (L. Bogni)

**Keywords:** viruses, hantavirus pulmonary syndrome, hantavirus, Argentina, Andes virus, research

## Abstract

We report a large case series of hantavirus pulmonary syndrome (HPS) in Argentina that was confirmed by laboratory restuls from 1995 through 2008. The geographic and temporal distribution of cases by age, sex, fatality rate, and risk factors for HPS was analyzed. A total of 710 cases were unequally distributed among 4 of the 5 Argentine regions. Different case-fatality rates were observed for each affected region, with a maximum rate of 40.5%. The male-to-female ratio for HPS case-patients was 3.7:1.0; the case-fatality rate was significantly higher for women. Agriculture-associated activities were most commonly reported as potential risk factors, especially among men of working age. Although HPS cases occurred predominantly in isolation, we identified 15 clusters in which strong relationships were observed between members, which suggests ongoing but limited person-to-person transmission.

## Abstract 2

TOC summary line: Limited person-to-person transmission is suggested.

Hantavirus pulmonary syndrome (HPS) was first recognized in 1993 during an outbreak of acute respiratory distress syndrome in the southwestern United States (1,2). Previously, only Old World hantaviruses had been associated with illness in humans as the causative agents of hemorrhagic fever with renal syndrome. After recognition of HPS, cases in other countries of Central and South America were quickly identified, along with the associated virus and rodent reservoirs (3–10).

Although serologic studies provided the initial evidence of hantavirus circulation in Argentina (11,12), the etiologic agent of HPS in Argentina was first described in 1995 after an outbreak occurred in the Andean sector of Patagonia where Andes virus (ANDV) was characterized (4). Several reports have been published since then, describing HPS cases in 4 regions of the country: Northwest, Northeast, Central, and Patagonia. Six lineages of ANDV were associated with HPS in the 4 regions of Argentina: AND-Oran, AND-Bermejo, AND-BsAs, AND-Lechiguanas, AND-Plata, and AND-South (10,13–16). Juquitiba virus (JUQV) and Laguna Negra (LN)–like virus were also found in the Northeast and Northwest regions, respectively (14,17).

We describe the epidemiologic features of a large proportion of confirmed HPS cases in Argentina. Detailed data were compiled for analysis of age, sex, onset of symptoms, clinical signs, case-fatality rates, geographic origin, and the most probable risk activities.

## Materials and Methods

### Study Site

**Figure 1:**
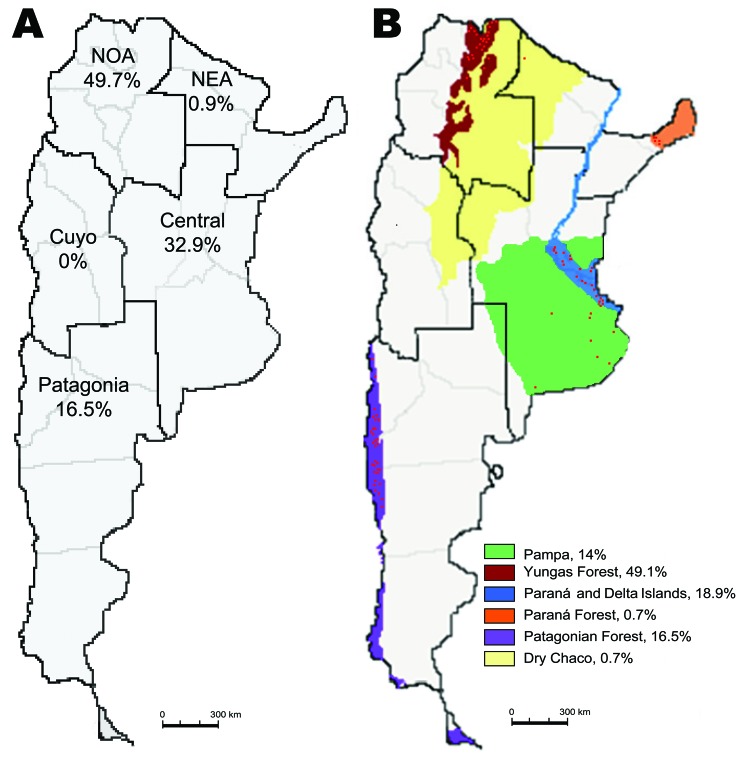
Distribution of hantavirus pulmonary syndrome (HPS) cases in Argentina, 1995–2008. A) The 5 Argentine epidemiologic regions and percentages of HPS cases in each one are shown. B) Six of the 18 ecoregions (18) represented by the colors indicated in the reference key; percentages of HPS cases in each ecoregion are shown. Location of HPS cases is represented approximately by point density. Total no. of cases analyzed: 692; confirmed cases of person-to-person transmission were excluded from this analysis. NOA, Northwest; NEA, Northeast.

Argentina is located at the southern extreme of South America. It has a large longitudinal extension, 3,779 km, and the highest altitudinal range of the continent. With a continental extension of 2,791,810 km^2^, Argentina is the second largest country in South America and the eighth largest in the world. The size of the country supports multiple natural ecosystems. Argentina has been divided into 5 epidemiologic regions: Northwest, Northeast, Cuyo, Central, and Patagonia (Figure 1). It has been also classified into 18 ecoregions on the basis of geographic, climatic, and biologic factors (18).

### Study Population

We analyzed suspected HPS cases from Argentina that occurred during 1995–2008 and for which samples were submitted to our laboratory for diagnostic confirmation. Standardized information was required for each suspected case (obtained by completion of a clinical/epidemiologic HPS form designed by the National Ministry of Health, Epidemiology Department). Samples were received from regional epidemiology units or directly from hospitals or private health systems. HPS diagnosis in Argentina during this period was performed in 2 validated national institutions: the Instituto Nacional de Enfermedades Virales Humanas (INEVH) and the Instituto Nacional de Enfermedades Infecciosas (INEI), components of the National Administration of Laboratories and Institutes of Health (ANLIS “Dr. C.G. Malbrán”). The selection of either of these 2 institutions was based on the convenience of the sender institution, without directions from national authorities, so samples were sent without distinction to either of them. We reviewed available data from all laboratory-confirmed HPS cases analyzed at INEI (n = 710), which represents 77.6% of the total cases submitted to the ANLIS “Dr. C. G. Malbrán” that fit the definition of laboratory-confirmed HPS cases. An acute febrile illness (>38.5°C) and any sign of respiratory compromise were required to meet the case definition of a suspected HPS case. The development of prodromal signs in contacts of previously confirmed HPS case-patients was enough to include them as suspected HPS case-patients.

### HPS Case Confirmation

Clinical diagnoses were confirmed if laboratory testing detected hantavirus-specific immunoglobulin (Ig) M or rising titers of hantavirus-specific IgG or detected viral genomic material in any tissue. Serum or whole blood samples were tested by ELISA for specific IgM (μ-capture technique) and IgG against ANDV as previously described (19). Viral RNA detection was performed by reverse transcription–PCR for the detection of the S and M segments, followed by nucleotide sequencing as previously described (10).

### Risk Factors

To analyze the type of exposure, we categorized information provided by 410 case-patients about risk activities during a 30-day period before illness: rural (persons who used to work in rural settings), wild (persons who performed activities in natural, nondisturbed, nonrural environments), peridomestic (rural or suburban residents without defined events of exposure in other places, considering housing and the surrounding land the source of infectious rodents), and undefined (persons with urban residence and no reported rural or recreational activities) (Table 1). Case-patients with a history of recent travel outside the country were excluded from the analysis (n = 12).

**Table 1:** Demographic characteristics of case-patients who had laboratory-confirmed HPS, by region, Argentina, 1995–2008*

Characteristic	North	Central	Patagonia	Northeast
No. case-patients (no. deaths)†				
Total	345 (59)	234 (72)	126 (51)	6
M	290 (45)	170 (49)	89 (33)	4
F	52 (14)	64 (23)	37 (18)	2
Case-fatality rate for all case-patients, %	17.1	30.8	40.5	0
Type of exposure				
Rural	111	34	22	1
Wild	34	20	17	1
Peridomestic	28	26	13	2
Contact with previous HPS case-patient	–	6	23	–
Other	25	30	6	–
Total	208	116	81	4

*HPS, hantavirus pulmonary syndrome; –, no reported events. †No. HPS cases in each region with confirmed diagnosis (n = 710). Type of exposure was classified on the basis of the most probable activities of risk (n = 410).

## Results

From 1995 through 2008, a total of 8,522 suspected HPS cases were submitted from the 5 Argentinean epidemiologic regions to our laboratory for diagnosis, and HPS was confirmed for 710 (8.3%). Prodromal symptoms did not differ from the HPS clinical picture previously reported (20–24); the prodromal phase was followed by different degrees of respiratory compromise, usually rapid and acute respiratory distress. However, 4 cases were confirmed in patients without respiratory manifestations. One of these cases was detected in the daughter of a woman with confirmed HPS during monitoring of the case-patient’s contacts. At the time of hospitalization, for all patients, platelet counts ranged from 15,000 to 305,000/mL (mean value 82,340; median value 68,000; n = 510); therefore, platelet count resulted to be an important laboratory indicator of a probable case of HPS.

Of the 710 HPS cases, 708 were laboratory confirmed by detection of IgM, and 2 were confirmed by detection of IgG and the viral genome by reverse transcription–PCR. Of the 708 IgM-positive samples, 606 (85.6%) were also IgG positive. Samples were received at the laboratory for diagnosis 11.6 days after onset of disease (mean value, n = 470), and definite laboratory-confirmed diagnosis was obtained within 2 days.

**Figure 2:**
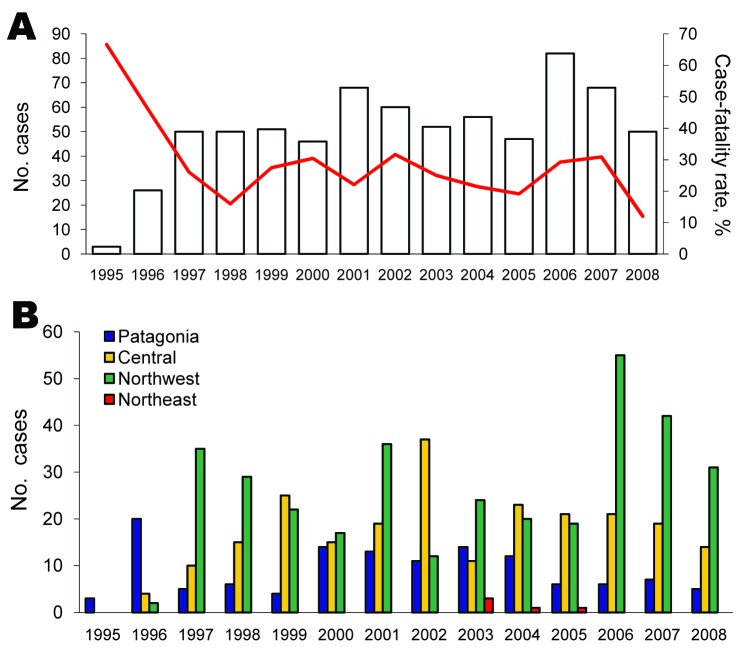
Annual hantavirus pulmonary syndrome case distribution and case-fatality rate, Argentina, 1995–2008. A) Annual case numbers (bars) and case-fatality rate (red line). B) Annual case distribution by region.

During the first 3-year period (1995–1997), the annual number of cases increased and then varied, ranging from 42 to 82 cases per year (Figure 2, panel A). Of the 710 confirmed cases, 183 deaths caused by HPS were reported, with an overall mortality rate of 25.8%. Case-fatality ratios declined during the first 4 years of the period (1995–1998) (χ^2^ test for trend, p = 0.0372).

An accurate analysis of the occurrence of HPS cases showed that their distribution was limited to small areas inside 4 of the 5 Argentine epidemiologic regions: Northwest, Northeast, Central, and Patagonia (Figure 1). The Northwest region accumulated the highest case number (49.7%), but in the Northeast region, only 6 cases have been confirmed since 2003 (0.87%). In the second most frequently affected region, the Central (32.9%), most cases occurred in Buenos Aires Province. In Patagonia, the affected area (16.5%), comprised the forested southern Andes Mountains. The southernmost case occurred >800 km from the disease-endemic area of Patagonia (25) (Figure 2, panel B). Comparative analysis of case distribution in epidemiologic regions and ecoregions showed that location of cases fit better within ecoregions. Cases occurred in 6 of the 18 Argentine ecoregions: Yungas Forest, Paraná Forest, Dry Chaco, Pampa, Paraná Delta and Islands, and Patagonian Forest (Figure 1, panel B).

**Figure 3:**
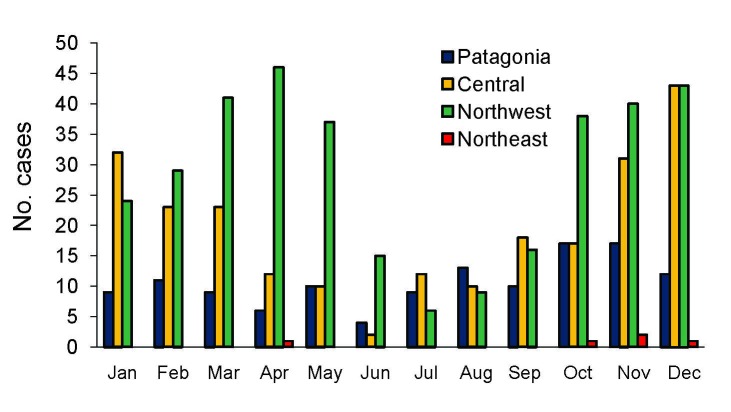
Hantavirus pulmonary syndrome case distribution, according to month of disease onset in disease-endemic regions, Argentina, 1995–2008.

Seasonal occurrence, determined on the basis of onset of symptoms and considering the 4 regions together, showed a decrease of cases only in winter (13.2%); a higher proportion of cases occurred in spring (35%), followed by summer (28.6%) and autumn (23.1%) (Figure 3). The highest peak occurred in autumn, mostly representing cases in the Northwest (64% of cases within the season in the Northwest region). Both the Northwest and Central regions showed similar peaks in spring (49% and 31%, respectively) and summer (43.4% and 42.4%, respectively). Although few cases occurred in the 2 remaining regions each month, a similar pattern could be inferred for both regions.

**Figure 4:**
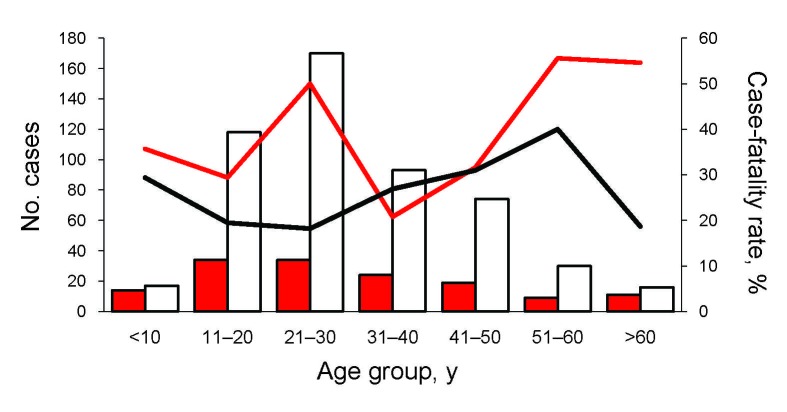
Hantavirus pulmonary syndrome cases and case-fatality rate, by age and sex distribution (n = 685), Argentina, 1995–2008. White bars and black line indicate male patients, red bars and line female patients.

Age range of case-patients was 0–77 years; mean age was 30 years and median was 28 years. Age frequency distribution in groups with a 10-year age range showed that the group of those 21–30 years accounted for the highest number of cases (Figure 4); 9.3% of cases were in children <14 years of age. The HPS-case population had a higher proportion of male patients than female patients in all 4 regions (78.8% vs. 21.2%; Table 1).

Analysis of case-fatality rates by region showed that values increased as the region’s location became more southern: Northwest (0%), Northeast (17.1%), Central (30.8%), and Patagonia (40.5%) (Table 1). Among these regions, the difference in case-fatality rates between the northern regions and those of the Central and Patagonia regions were significant (χ^2^; p = 0.0001 and p<0.0001). However, the difference among the Central and Patagonia regions was not significant (χ^2^; p = 0.064).

The case-fatality rate was significantly higher for female than for male patients: 34% versus 21% (χ^2^; p = 0.0013); it was also higher for patients without an IgG response than for the IgG-positive group, 51% versus 21% (χ^2^; p<0.0001), whereas no difference was found between the elapsed time from symptom onset to sample collection among patients with fatal and nonfatal cases (mean 5.28 and 5.78, respectively; p<0.093 by *t* test). This analysis was performed with >572 acute-phase samples.

In the rural category, the most frequently reported activities were agriculture associated or general work on farms, mainly preparing land for cultivation, clearing weeds, planting and harvesting of field crops, and cleaning out barns or other outbuildings. The wild category included case-patients with probable exposures in natural environments during recreational activities, tourism, or activities related to occupations such as sanitary agent, security personnel, or truck driver. The peridomestic category included primarily housewives and children.

In regard to particular activities of risk, only 129 (32.8%) of 393 case-patients reported a defined event of exposure. The most frequently reported activities were the following: occasional recreational activities in wild areas, cleaning outbuildings in rural areas, and having contact with an HPS case-patient. Thirty-five case-patients had recent contact with a previously confirmed HPS case-patient. Among these, the suspicion of human-to-human transmission was based on the patient’s close relationships with other case-patients, the lack of risk or low risk in other activities, and on the length of time that had elapsed between the onset of symptoms in both related case-patients.

The main indicator that raises suspicion of human-to-human transmission was a long period before symptom onset for both case-patients. In previous studies, based on confirmed cases of person-to-person transmission, the incubation period ranged from 12 to 27 days (10), which suggested that viral spread likely occurred near the end of the prodromal phase (26). Considering the length of the prodromal phase, a difference in time of symptom onset between 2 related case-patients (16–35 days) is used to raise suspicion. Case-patients who are exposed to the same permanent infectious source are not considered to demonstrate human-to-human transmission. To differentiate human-to-human transmission from common exposure to infectious rodents, after an accurate epidemiologic investigation, comparing long viral nucleotide sequences from patients has been useful (10,26). Fifteen of 35 clustered cases corresponded to a unique cluster in which person-to-person transmission was first demonstrated (27). The remaining 20 cases belonged to 14 additional clusters (Table 2). Nine clusters occurred in Patagonia, 5 in the Central region, and the remaining cluster had cases from both regions. We have previously confirmed person-to-person transmission in 2 of these 14 clusters (26,27).

**Table 2:** Epidemiologic characteristics of clustered HPS cases, Argentina, 1995–2008*

Cluster no.	Contact case-patient no.	Region	Case-patient sex/age, y		Relationship with index case-patient	Ambient risk†	Days between symptom onsets
			Index	Contact			
1‡	C1-s	Central	M/41	M/14	Son	No	27
2	C2-d2	Central	F/12	F/11	Sister	High	4
	C2-s	Central	F/12	M/ND	Brother	High	ND
	C2-m	Central	F/12	F/40	Mother	High	12
3	C3–2	Central	M/28	M/27	Friend, roommate	High	2
	C3–3	Central	M/28	M/21	Friend, roommate	High	15
	C3–4	Central	M/28	M/30	Friend, roommate	High	19
4‡	C4-b	Central/ Patagonia	M/39	M/58	Friend, roommate	No	15
	C4-c	Central	M/39	M/39	Friend, roommate	No	23
5	BA04–2	Central	F/24	F/0	Daughter	ND	28
6	BA06–2	Central	M/53	F/28	Wife	High	31
7	NQ00–2	Patagonia	M/46	M/10	Son	Low	22
8	NQ01–2	Patagonia	M/40	F/44	Ex-wife	No	22
9	NQ06–2	Patagonia	M/48	F/53	Wife	Low	30
10	RN95–2	Patagonia	M/38	F/25	Girlfriend	No	21
	RN95–3	Patagonia	M/38	F/15	Daughter	No	29
11	RN00–2	Patagonia	F/25	M/64	Husband	Low	20
12	RN03–2	Patagonia	M/31	F/28	Wife	Low	40
13	CH00–2	Patagonia	F/36	F/3	Daughter	Low	–
14	CH00–4	Patagonia	M/28	F/24	Wife	Low	22

*HPS, hantavirus pulmonary syndrome; ND, not determined; –, secondary case-patient did not show symptoms of illness. †Ambiental risk was classified in as follows: no, without ambient risk, urban residence; low, rural residence and no evident rodent exposure; high, rural residence and evident rodent exposure. ‡Clusters with confirmed person-to-person transmission.

## Discussion

This report summarizes epidemiologic characteristics of 77.6% of HPS cases in Argentina since the disease was detected. The considerable extension, especially in the longitudinal direction, and multiple natural settings represented a great limitation to collecting the information and designing and conducting an epidemiologic study. Most of the data presented here were collected as part of local routine surveillance or emergency outbreak investigations without a consensus strategy or general design. HPS has affected 4 of 5 Argentine epidemiologic regions (Cuyo is the only region without reported cases) (Figure 1, panel A). This finding was consistent with the report of National Ministry of Health (www.msal.gob.ar/htm/site/sala_situacion/PANELES/boletines/bepAnual/BEPanual2006_Zoonosis.pdf). The Northwest and Central regions were the most affected. The distribution of HPS cases fit better inside ecoregions rather than inside epidemiologic regions, so the classification of the country in ecoregions could be more appropriate for understanding HPS occurrence (Figure 1, panel B). In northern Argentina, most of the cases grouped in the Yungas Forest (49.1%). Few cases occurred in Dry Chaco and Paraná Forest (0.7% each). In the Central region, cases accumulated in Humid Pampa (14%) and in Paraná Delta and Islands (18.1%), mainly in large suburbs. With regard to Patagonia, HPS cases accumulated in the cooler, high, Andean forested zone, a narrow strip along the Andes Mountains, which corresponded with the Patagonian Forest (16.5%). Each ecoregion is formed by particular ecosystems with specific plant and animal species especially adapted to them. Because the present analysis does not represent all of the registered cases in the country (22.4% were diagnosed in the other national institution), we recognize this as a limitation of our work. We consider that the real proportion of reported cases in each of the epidemiologic regions, as well as other epidemiologic factors included in this descriptive study, could differ slightly.

Since the first description of HPS in southwestern Argentina, new cases have been reported, and new disease-endemic areas have been gradually recognized. However, the current distribution of HPS cases does not imply that no other areas are affected. Samples from patients with suspected HPS were submitted mainly from the disease-endemic areas, but we also received samples from other areas. Among these, we confirmed a few HPS cases, which illustrates that new areas are potentially disease endemic. This is the case for the Dry Chaco region. Recently, we confirmed 1 case from the northern Paraná Delta and Islands ecoregion; the patient had no history of travel outside the area of residence. In Patagonia, outside the Patagonian Forest, several seroprevalence studies have been conducted in rodent populations in which no hantavirus-positive animals were found (25,28,29). We also received samples from suspected case-patients from this area, and no HPS cases were confirmed. The situation of the Cuyo region is less clear because few samples from persons with suspected HPS and few rodents were analyzed. Thus, future change in HPS case distribution might be expected.

HPS is a zoonotic disease, and its distribution is expected to be similar to that of its rodent reservoirs. In northwest Argentina, 3 pathogenic hantaviruses, AND-Orán and AND-Bermejo lineages and LN-like-virus, have been associated with *Oligoryzomys longicaudatus*, *O. chacoensis,* and *Calomys callosus* rodents*,* respectively (13,15,17). Inside the Paraná Forest ecoregion, AND-Lechiguanas was characterized from 1 HPS case, JUQV was characterized from 2 HPS cases, and *O. nigripes* rats were identified as the reservoir species of JUQV in the area (14). Three pathogenic lineages of ANDV have been identified in the Central region, AND-BsAs, AND-Lechiguanas, and AND-Plata (10,16,26). *O. flavescens* rice rats have been identified as the reservoir of AND-Lechiguanas and AND-Plata (13,30). In Patagonian Forest *O. longicaudatus* is the principal reservoir of AND-South lineage (13,28). Future studies will be necessary to determine the rodent reservoirs in Dry Chaco in both epidemiologic regions, Northwest and Northeast.

The increasing number of HPS cases during the first 3 years of the study period could be attributable to an improvement in detection of suspected cases. Cumulative frequency analysis showed that cases in the whole country occurred throughout the year; the highest number occurred during spring/summer. The case-fatality rate decreased during the first years of the period analyzed; the decrease could be attributed to the medical experience acquired during clinical treatment, to an earlier recognition of suspected cases, especially in disease-endemic areas, and to recognition of less severe cases. Currently, the clinical picture for a suspected HPS case is not necessarily associated with a severe respiratory disease. However, differences were observed in virus lethality between each Argentine region; the proportion of deaths increased as the region considered became more southern. The high case-fatality rate in Patagonia could be associated with higher viral load input because of climatic conditions that supported the maintenance of aerosolized infectious virus. However, multiple factors such as different rodent reservoirs and different lineages in each region could influence the proportion of deaths. Another possibility is that these differences could be because fewer mild cases are detected in the Patagonia and Central regions. Future research will be necessary to assess whether these or other factors could be considered predictors of the likelihood of death.

Humoral immune response seems to be early and strong in HPS case-patients from South America; however, low IgG titers or the absence of IgG has been associated with a higher mortality rate (10). In that study (10), by means of a stratified analysis in 91 cases, we showed the usefulness of IgG titers as a predictor of outcomes. In the present study, we confirmed that IgG titer was a strong predictor of outcomes for 572 case-patients in Argentina. Similar observations were described for HPS caused by Sin Nombre virus infections in 2 studies of 26 case-patients (31) and, more recently, in 51 HPS case-patients (32).

Changes in natural ecosystems have altered the abundance and distribution of rodent species and might have favored colonization of agro-ecosystems by hantavirus reservoir species (25). All 5 affected Argentine ecoregions have different degrees of anthropogenic disturbances (33). The Yungas Forest and the Pampas are the most disturbed ecoregions. Future studies will be necessary to find factors associated with HPS emergence in each region, such as anthropogenic disturbance, population density, etc.

HPS is strongly associated with rural activities, which are performed mainly by men of working age. This is the probable reason for the predominance of young men among HPS patients. Although agricultural activities seem to be the main cause of exposure for men, peridomestic exposures were most frequently reported for women and children. Contact with rodents is extremely common in many rural locations. Clusters of cases were rare in general and usually more frequent in the Patagonia region, but in the Central region, several clustered cases were reported. Human-to-human transmission was previously confirmed on the basis of strong epidemiologic and genetic evidence (26,27). For other cases, secondary cases after the index case were classified as suspected of having person-to-person transmission because such transmission could not be either confirmed or rejected. Two ANDV lineages have previously been identified in events of human-to-human spread: AND-South and AND-BsAs lineages (26,27). Confirmed instances of person-to-person transmission represent 2.5% of the total case number during 1995–2008. Future events or outbreaks of person-to-person transmission would be expected, especially in the Patagonia and Central regions.

The reason the death rate is higher for women is a matter of speculation. It is well established that the sex of a host can significantly affect susceptibility to infections with several pathogens. Differences in male and female immune responses have been recognized for some time. Gender-determined differences in susceptibility to virus infections have been reported for encephalomyocarditis virus (34), vesicular stomatitis virus (35), and coxsackievirus B3 (36). Gender-dependent differences in plasma cytokine responses have been found in patients infected with Puumala virus and Old World hantavirus (37). Further studies will be necessary to determine whether different cytokine profiles or other immune factors could explain the higher proportion of deaths in female patients with HPS.

The present analysis of HPS in Argentina contributes to an understanding of its distribution and transmission. Although HPS is a relatively rare disease, it is among the most pathogenic of human viral infections. As more cases are recognized and risk factors are better identified, it will be possible to enhance surveillance efforts and to evaluate prevention measures for HPS.
